# Development and validation of a nutritional and nursing risk assessment method for diabetic patients

**DOI:** 10.3892/etm.2014.1575

**Published:** 2014-02-24

**Authors:** JING WANG, YUN LIN, KAIHONG ZENG, YONGTAO YANG, XUEFEI HU, RONG ZHAO, ZEYUE LI

**Affiliations:** Department of Nutrition, Sichuan Academy of Medical Sciences and Sichuan Provincial People’s Hospital, Qingyang, Chengdu, Sichuan 610072, P.R. China

**Keywords:** diabetes, nutritional risk assessment, risk management, nursing risk

## Abstract

The present study aimed to develop and evaluate a nutritional and nursing risk assessment method for diabetic inpatients to improve healthcare and risk management. Diabetic inpatients diagnosed according to the World Health Organization guidelines, together with their nursing staff, were divided into two groups for nutritional and nursing risk assessment. Data from one group were used to establish the assessment method, and data from the other group were used to evaluate the reliability and effectiveness of the method. To establish the method, various risk variables in the nutritional and nursing processes were evaluated by logistic regression analysis; the score and probability of the risk variables were determined based on odds ratios. The overall nutritional and nursing risk for individual inpatients was then judged by the accumulated scores. The analysis showed that there were a number of risk factors, including age and body mass index. The risk was shown to increase with increasing score for the inpatients, and the χ^2^ test (P<0.01) was used to indicate a significant association. When the score was 50, the sensitivity and specificity of the method used to detect the nutritional and nursing risk were 88.3 and 66.5%, respectively, with predictive positive and negative rates of 12.83 and 98.53%, respectively. Therefore, the method is simple, cost-effective and fast; it can be used to screen a large number of patients by nursing staff and can also be used by patients themselves. Overall, the method is an effective and practicable nutritional and nursing risk assessment and educational tool.

## Introduction

In recent years, with increasing public health knowledge, liability issues and awareness of self-protection, medical healthcare providers have faced increasing risks. Delivering safe nursing and identifying and reducing risks have become the main tasks for nursing staff. Neuroma studies have been carried out on nursing risks and risk management (1–3), resulting in a number of risk analysis and management systems, particularly for important clinical departments and major common diseases (4–6). These include analyses of the types of risks and their causes and management. For diabetes, a number of risk assessment methods are available, including nutritional risk screening, which was designed to identify diabetic patients through assessment of their nutritional condition (7–9). However, little has been reported on an integrated nutritional and nursing risk assessment from the patient’s and the nursing staff’s prospective. The present study was conducted to establish and evaluate a risk assessment method for diabetic inpatients in order to enable nursing staff and patients to predict risk factors for nutritional intervention.

## Subjects and methods

### Subjects

The data of 2,065 participants were selected from the type II diabetic inpatients in the Departments of Elderly Endocrinology and Endocrinology at the Provincial People’s hospital between January 2009 and December 2012, together with data for their nursing attendants. Subjects with incomplete data were excluded. The participants consisted of 1,276 males and 786 females, aged between 60 and 98 years old, with an average age of 68±11.32 years old. The participants were grouped randomly into two groups, A and B, consisting of 1,043 and 1,033 participants, respectively. The two groups had comparable profiles, including age and gender. This study was approved by the Sichuan Academy of Medical Sciences Review Board (Institutional Review Board no. B4589888).

### Questionnaire for risk assessment

The risk assessment questionnaire was designed to have two parts, one was designed to review the nursing risk for nursing staff and the other was designed to evaluate the risk for nutritional intervention. Part one was used to assess the nursing staff for their knowledge about the disease, physical exercise, the functions and side-effects of antidiabetic drugs, and the recording and monitoring of patient’s weight, blood sugar level and diet. The rationality of the staff’s guidance for balanced patient food intake and their knowledge of a non-indicative label for hypoglycemic drugs were also assessed.

The second part was to assess the patient’s personal and nutritional risk factors, including gender, age, height, weight, administration of antidiabetic drugs, daily intake of staple food, intake of vegetables and fruits, diet composition (such as high fat and low fiber), dining time (fixed or not), dining habit (whether the patient dined out more than three times per week) and exercise (whether the patient was sitting down whilst working, without much movement, and whether the patient exercised for 3–4 h per week). Other information that was collected in this section of the questionnaire included any family history of diabetes (namely, if the patient’s parents or siblings were diabetic), smoking and drinking histories, a history of high blood sugar level (whether or not the patient had previously been diabetic or was a latent diabetic) and a history of any hypertension, hyperlipidemia and coronary heart disease.

### Methods

Prior to conducting this investigation, consent was obtained from the clinicians, with whom the projects were discussed, and who provided support. In total, 15 trained nutrition nurses, six with excellent accuracy in completing questionnaires, were selected to participate in this investigation. All participants were asked to sign their informed consent and complete the questionnaire under the supervision of the trained nurses.

Blood sugar level change was used as a dependent variable; if it changed, it was assigned a value of 1, and otherwise a value of 0. All other factors were treated as independent variables, including age and gender. These variables were used in an unconditional multivariate logistic regression analysis to screen for risk factors. A factor (Xi) with P<0.05 was considered to be significant. In the logistic regression analysis, regression coefficient β indicates the relative degree of risk increase from increments of the independent variable. Therefore, values that were 10 times that of the β value were used to calculate the risk score of the respective variables. An individual’s predicted nursing risk was, therefore, determined by the number and score of the risks that they possessed.

The risks and their scores that were determined using the data from group A were used to evaluate group B in order to validate the method. The accumulated score [∑(Xi × Score)] of individuals in group B was calculated. The cutoff value for a high-risk patient was determined and the diagnostic accuracy of the method was tested by Youden’s index (10). The 2006 World Health Organization diagnostic criteria of diabetes was used to measure the level of blood sugar (11). The blood sugar levels were considered changed if there was either a ≥7.0 mmol/l difference in fasting plasma glucose or an 11.1 mmol/l difference in post-challenge 2-h plasma glucose.

### Statistical analysis

Unconditional multivariate logistic regression analysis was used to analyze the correlation between the variables and blood sugar level change. Percentage values were tested for any differences using the χ^2^ test. The area under the receiver operating characteristic (ROC) curve (AUC) was used to calculate the risk of developing diabetes. The data were processed using SPSS software (version 16.0; SPSS, Inc., Chicago, IL, USA), and the statistical significance was indicated by P<0.05.

## Results

### Identification of risk factors

To identify the risk factors, data from group A were analyzed using unconditional multivariate logistic regression analysis, with blood sugar level change used as a dependent variable.

Among the 20 variables analyzed, eight were found to be significantly associated with the blood sugar level change (P<0.05; Table I), including age, body mass index, waist to hip ratio, diet and histories of smoking, alcohol consumption, diabetes and high blood pressure. The coefficient β of the variables was multiplied 10 times and used as the risk score (Table I).

### Risk scores of nutritional and nursing variables

To evaluate the level of risk, the accumulated scores of the identified significant variables, as well as the blood sugar level change over the accumulated scores were calculated for individual patients (Table II). As shown in Table II, the change in the blood sugar level increased with the accumulated scores, with a significant association between them (P<0.001). Therefore, the score can be used to differentiate between patients with different levels of risk. Such differentiation would aid further screening of high-risk patients and the implementation of proper preventive measures.

### Evolution of the nutritional and nursing risk assessment

The patients in group B were evaluated at different accumulated scores, used as cutoff values, and the results are shown in Table II. It was found that Youden’s index was at the maximum when the score was 50. Therefore, the score was used as the cutoff value. At this point, the sensitivity and specificity were 88.3 and 66.5%, respectively. This means that if 34% of the high-risk inpatients are screened, 3/5 of the nursing risks can be identified by this method in the same inpatient population, which has predictive positive and negative rates of 12.83 and 98.52%, respectively.

**Figure 1:**
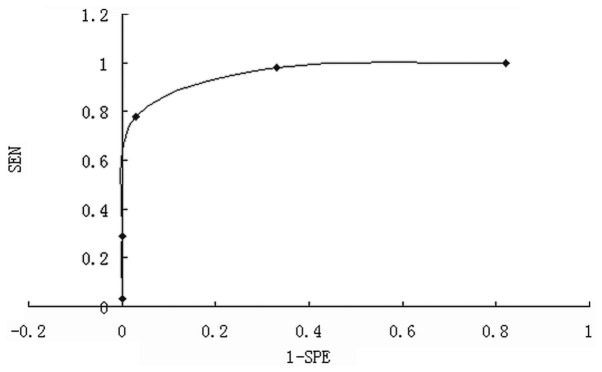
ROC curve for the nutritional and nursing risk assessment to confirm method reliability. ROC, receiver operating characteristic; SEN, sensitivity; SPE, specificity.

The reliability of the method was further confirmed by ROC curve analysis, where the AUC was 0.82, with a standard error of 0.018 and a 95% confidence interval value of 0.783–0.856 (Fig. 1), indicating that the risk assessment method is able to reliably identify the risks in diabetic patient care.

## Discussion

Diabetes is a common metabolic endocrine disease, often accompanied by systemic chronic complications in multiple organs and systems. Diabetes has high morbidity and mortality rates, resulting in a physically and economically heavy burden on patients. According to the 2007–2008 statistics, the overall prevalence of diabetes has reached 9.7% in China (11,12). There is an urgent requirement to perform a risk assessment for the large number of patients with diabetes in order to improve their care and treatment. If such assessments can be made from a nursing prospective to identify the nutritional risk at an early stage, it would aid in providing improved nutrition-based therapy and early warning signs for healthcare activities.

A number of risk predictive methods are available to determine whether there are nutritional risks for diabetic patients, and what the clinical outcomes for the patients with these risks would be (13–15). These methods have been used as preliminary tools to screen for individuals with a high risk of diabetes, and are therefore useful in assisting and identifying the risk of diabetes in patients at an early stage. The method described in the present study is an integrated screen that may be used by nursing staff and patients to assess nutrition and nursing-related risks. This would consequently result in nursing staff having an improved understanding of the patient’s nutritional care requirements, in order to provide superior educational and healthcare service to patients and to prevent or delay disease progression, with fewer complications.

Results from this study demonstrated that nutritional and nursing risk assessment screening can effectively reduce the nursing risk and should be part of the integrated risk management system in hospitals. This method can be used by nursing staff and patients. Risk assessment screening is a simple, fast and highly sensitive method, with a high detection rate for positively diagnosed patients and a low implementation cost and false positive (or high specificity) rate.

## Figures and Tables

**Table I:** Logistic regression analysis of risk factors.

Variable	β	OR	95% CI	P-value
Age	1.826	6.379	2.911–14.480	<0.05
BMI	1.858	7.394	3.384–16.190	<0.05
WHR	1.011	2.844	1.295–4.468	<0.05
Diet	2.004	2.931	1.499–4.321	<0.05
Smoking history	2.938	3.542	1.639–5.707	<0.05
Alcohol consumption	2.036	2.212	1.162–3.933	<0.05
History of family diabetes	1.868	5.740	3.814–8.933	<0.05
History of high blood pressure	2.048	15.616	8.991–29.340	<0.05

OR, odds ratio, CI, confidence interval; BMI, body mass index; WHR, waist to hip ratio.

**Table II:** Nutritional and nursing risks for diabetic patients measured by the accumulated scores.

Accumulated score	No. of patients	No. of patients with blood sugar change^a^	Patients with blood sugar change, %	OR
0–25	173	1	0.58	1.00
26–50	484	11	2.27	4.18
51–75	317	29	9.15	15.70
76–100	43	15	34.88	92.28
101–125	5	4	80.00	738.94

a Blood sugar levels were considered changed if there was either a ≥7.0 mmol/l difference in fasting plasma glucose or an 11.1 mmol/l difference in post-challenge 2-h plasma glucose levels. OR, odds ratio.
